# Hypersensitivity pneumonitis: lessons for diagnosis and treatment of a rare entity in children

**DOI:** 10.1186/1750-1172-8-121

**Published:** 2013-08-08

**Authors:** Matthias Griese, Melanie Haug, Dominik Hartl, Veronika Teusch, Judith Glöckner-Pagel, Frank Brasch

**Affiliations:** 1 Dr. von Haunersches Kinderspital, University of Munich, Member of the German Center for Lung Research, Lindwurmstr. 4a, D-80337 Munich, Germany; 2 Department of Pediatrics, University of Tuebingen, Tuebingen, Germany; 3 Institute for Pathology, Bielefeld, Germany

**Keywords:** Biopsy, Bronchoalveolar lavage, Children, Diffuse parenchymal lung diseases, Exogenous allergic alveolitis = extrinsic allergic alveolitis, Precipitins, Steroid treatment

## Abstract

Hypersensitivity pneumonitis (HP) also called exogenous allergic alveolitis = extrinsic allergic alveolitis in children is an uncommon condition and may not be recognized and treated appropriately.

To assess current means of diagnosis and therapy and compare this to recommendations, we used the Surveillance Unit for Rare Paediatric Disorders (ESPED) to identify incident cases of HP in Germany during 2005/6. In addition, cases of HP reported for reference from all over Germany to our center in the consecutive year were included.

Twenty-three children with confirmed pediatric HP were identified. All (age 9.4 y (4.4-15.1) presented with dyspnoea at rest or with exercise, mean FVC was 39% of predicted, seven of the 23 children already had a chronic disease state at presentation. IgG against bird was elevated in 20, and against fungi in 15. Bronchoalveolar lavage was done in 18 subjects (41% lymphocytes, CD4/CD8 1.99), and lung biopsy in 6. Except 2, all children were treated with prolonged courses of systemic steroids. Outcome was not favourable in all cases.

Late diagnosis in up to a quarter of the children with HP and inappropriate steroid treatment must be overcome to improve management of HP. Inclusion of children with HP into international, web-based registry studies will help to study and follow up such rare lung diseases.

## Introduction

Hypersensitivity pneumonitis (HP) - in Europe called extrinsic or exogenous allergic alveolitis (EAA) - is a complex syndrome incited by numerous inhaled agents including agricultural dusts, bio-aerosols, fungal-, bacterial- or protozoan microorganisms, and certain reactive chemicals. In children it is a relatively uncommon condition and the two major inciting allergens are bird (avian) allergens including down and inhaled particles derived from fungi, like thermophilic actinomycetes, or rarely fusarium [1], aureobasidium [2,3] and epicoccum [4].

A previous NHLBI/ORD Workshop has summarized state of the art and the needs and opportunities for research in HP [5]. It was stated that because pediatric cases of HP are rarely recognized or reported, knowledge is limited and is based mostly on case reports and small series of patients. Between 1960 and 2005, 95 cases of HP in children have been reported in the literature [6-8]. In contrast to the data from adults, 95% of the cases were males and 25% had a family history of HP [5]. This hints to some reporting bias. The finding of clubbing in 31% (10/32) of the children, suggested that in the past the disease was recognized late in its clinical course. Importantly, as deaths from HP have been reported in children as well as adults [5,6], treatment may be more difficult than anticipated. This is highlighted by the fact that 3% of the children were not treated with removal from the exposure. Also, treatment with corticosteroids is very controversial. In addition to oral long term therapy most frequently done (about 66%) [5], methylprednisolone pulse therapy [9,10] or inhaled budesonide [11] were suggested. Current recommendations in adults, as well as our personal practice in children, clearly suggest no steroid treatment at all, if possible [12].

Due to its rarity, many paediatricians and general practitioners are likely not to be very familiar with the clinical presentation and diagnosis of HP, and many pediatric pulmonologists may not use up to date treatment. All these issues, including an unknown proportion of cases of interstitial lung disease in children which may represent undiagnosed HP, suggested that current information on the diagnosis and treatment of HP is warranted. We used a survey that we performed to determine the incidence of pediatric diffuse parenchymal lung diseases in Germany, to learn more about the current status of HP in children in Germany [13]. The goal of this study was to assess current means of diagnosis and therapy of HP, to compare this to the recommendations and to propose ways to improve future management with the help innovative strategies for rare lung diseases.

## Methods

### Study subjects and study design

The German Surveillance Unit for Rare Paediatric Disorders [Erhebungseinheit für seltene pädiatrische Erkrankungen in Deutschland (ESPED)] sends out monthly inquiries to all paediatric hospitals, to report specific conditions investigated prospectively. We used this system from 2005 to 2006 to monitor interstitial lung diseases [13]; the number of children under 17 years of age under surveillance was 14 393 400. The overall return rate was 97%, in case of a positive reply, a detailed questionnaire was sent to the reporting institution. We identified 11 children with HP during this 2 year period. 12 additional children who were reported to us for reference in the following year 2007 from all over Germany were also included. Only newly diagnosed cases of pediatric HP were eligible for the study. The main study objective was to describe current diagnosis and treatment of HP and to compare this practice to recommendations [12]. The surveillance study and the retrospective analysis of the children with HP were approved by the local ethical committee (EK 355/04 and letter 2010-3-5) and written informed consent of the subjects and parents or legal guardians was obtained.

The surveillance study was prospective; retrieval of detailed information on verified cases was retrospective. Clinical information at diagnosis and from follow –up was collected from all subjects. Of the 23, 22 were old enough for lung function measurements, 17 underwent bronchoscopy including BAL with cytology, and 13 chest CTs were done. Diagnostic open lung biopsies were done in 6 of the patients. None of the patients were treated with systemic corticosteroids therapy at the time of the diagnosis. All children had definite diagnosis of HP, based on the criteria proposed by Schuyler et al. 1997 [14].

### Serum and bronchoalveolar lavage fluid analysis

In all children serum IgG level for fungal and bird allergens and in 17 children mycoplasma serology were determined and the results judged as normal or increased according to the appropriate lab reference values. BAL was performed during flexible bronchoscopy. Lavage sites were middle or lower lobes with no preference for radiological more affected areas. Four times 1 ml per kg body weight of sterile 0.9% saline solution were instilled and then aspired [15]. The pooled fluid from 2^nd^ to 4^th^ aspirations were processed for differential cell counts, and stained according to May-Grünwald. In 7 children CD4+ and CD8+ lymphocytes were determined by flow cytometric investigation.

### Chest x-ray and CT scoring

We evaluated the radiographs and computed topographies (CT) of the chest from each patient independently and blinded, knowing nothing about the patients, the severity of their diseases, and the chronological sequences of the images. First we assessed the quality of the scans and differentiated between high-resolution and multi-slice CT examinations with or without contrast medium. Afterwards we evaluated on both modalities, if the hilar lymph nodes were enlarged (yes/no). Then we divided each lung into three parts. The apical zone was defined as the area from the apex of the lung to the tracheal bifurcation, the middle zone from the tracheal bifurcation to the lower pulmonary vein, and the inferior zone from the lower pulmonary vein to the diaphragm. For each zone on both sides we evaluated on every plain film as well as on each CT scan different pathologies with a scoring system. “0” meant, the pathology was absent, “1” represented mild changes, “2” stood for medium abnormalities, and “3” for massive findings. We scored presence and extent of linear, reticular, or nodular patterns, cysts, bronchiectasis, ground glass opacities, emphysema, and consolidations. The pathologies were defined according to the Fleischner society [16].

### Lung biopsy

Individual slides of all surgical lung biopsy specimens were stained with hematoxylin and eosin, Prussian blue (iron stain), Periodic acid-Schiff reaction (PAS) stain (Glycogen, neutral mucopolysaccharides), and van Gieson’s Stain (which demonstrates differential staining of collagenized connective tissue, smooth muscle and elastic tissue) and were assessed in a blinded manner by a pediatric pathologist (FB). Furthermore, immunohistochemical stains for T-lymphocytes (CD3), B-lymphocytes (CD20), and macrophages (CD68) were performed.

### Diagnosis of HP

The diagnostic criteria for HP included a known exposure to an offending antigen, the presence of specific IgG antibodies in serum against the identified antigen, compatible clinical, radiographic, or physiologic findings, a bronchoalveolar lavage (BAL) with lymphocytosis and in some cases a histopathology showing poorly formed, non-caseating granulomas or mononuclear cell infiltrates.

### Statistical analysis

The results are reported as mean ± standard deviation (SD) or frequencies of patients expressing a particular feature or not. For comparison of the frequencies the Fisher exact test was used. A two sided p-value of < 0.05 was considered significant. All individual data are available in the Additional file 1.

## Results

### Clinical presentation

During a period of 3 years 23 cases in children with the confirmed diagnosis of HP were collected from all over Germany. Mean age at diagnosis was 10 y. The children presented with acute (a, n = 6; symptoms prompted assessments within days to one week), subacute (s, n = 8; symptoms for at least one week, but less than 4 weeks duration), and chronic (c, n = 9, symptoms for longer than 1 month) disease expression. The time to diagnosis after presentation to a physician was brief, however 3 of 23 children already had clubbing, suggesting that in some cases it may have taken more time to see a physician. The children were generally sick, with chronic cough, dyspnoea at rest, cyanosis, and a significant weight loss of up to 3 to 4 kg of body weight. The responsible antigen by history were bird or downy feathers alone in 16 of 23 cases, fungus alone in 3 of the 23 cases, both in 3 cases and in one the antigen could not be suggested from history (Table 1 and Additional file 1: Table S1).

**Table 1:** Baseline data of the 23 children with hypersensitivity pneumonitis included into the study

	
Sex	9 male of 23 total
Age at 1^st^ visit (y)	9.8 ± 3
Time to diagnosis (mon)	1.3 ± 1
Initial presentation	
Chronic cough, dyspnoea at rest, cyanosis, clubbing	15, 13, 11, 3 of 23
Loss of weight per week until diagnosis (kg)	- 0.73 ± 0.49
Non-pulmonary diagnoses	Atopic eczema (2), Diabetes mellitus Type I (1), hyperthyreoiditis, adipositas (4), house dust mite allergy (2), bronchial asthma (1), celiac disease (2), small stature (1), hypothyroidism (1), alopecia areata (1), enuresis nocturna (1), vitiligo (1), GERD and nissen fundoplication (1)
Serum measurements	
Elevated fungus IgG	17 of 23
Elevated bird IgG	21 of 23
Total IgG (fold upper limit)	1.2 ± 0.6
LDH i. S. (U/l)	352 ± 189
ACE i. S.(U/l)	53 ± 31
Positive serology for mycoplasma	9 of 17 assessed
Bronchoalveolar lavage measurements	Done in 17 children
Total cell count (/μl)	8800 ± 11017
Macrophages (%)	41 ± 25
Lymphocytes (%)	46 ± 26
Neutrophils (%)	13 ± 13
Eosinophils (%)	2.6 ± 2.1
CD4+/CD8+ (% Lymph)	36 ± 16/48 ± 25
CD4/CD8 Ratio	2.0 ± 2.8
Cultured bacteria	None
Mycoplasma/Chlamydia	7 negative of 7 assessed for PCR in BAL
Lung function measurement	Done in 22 children
FEV1 (% pred)	44 ± 21
FVC (% pred)	38 ± 15
MEF25 (% pred)	62 ± 45
DLCOcHb (% pred)	52 ± 28
SaO2 (%) Rest/excercise	93 ± 27/83 ± 40
pO2 (mmHg) Rest/excercise	64 ± 34/57 ± 20
pCO2 (mmHg) Rest/excercise	38 ± 20/34 ± 12
Lung biopsy	Done in 6 children

Data are given as absolute numbers or mean ± SD.

### Laboratory results

In contrast to the type of exposure obtained from history, 15 children had elevated IgG antibodies against both, fungus and birds or downy feathers. Only 5 of the 15 children with a history suggestive of a reaction against birds or downy feathers had only these antibodies, and only 1 of the 3 with a history suggestive of fungus, had solely antibodies against fungus. Thus the specificity of elevated IgG antibodies is very low (Table 1 and Additional file 1: Table S2). Total serum IgG was not significantly elevated (expected mean 1.0, actual mean 1.18; discrepancy −0.18, 95% CI of discrepancy −0.026 to 0.38, P = 0.08, one sample t test). Of interest, in 16 of 23 children an atypical pneumonia was suspected and treated with macrolides or tetracycline, serology was positive for mycoplasma in 9 of 17 children tested, whereas PCR in BAL was negative in all tested cases.

### BAL results

Total cell count was elevated in some, but not all cases, whereas the cell differential showed a lymphocytosis in 91% (21 of 23). In 2 children neutrophilia was dominant, indicating the acute phase; in one of these lung biopsy confirmed the diagnosis of HP (Additional file 1: Table S3). The ratio of CD4/CD8 positive lymphocytes was elevated on average, however associated with a relatively large, well known scatter [8].

### Lung function

All children had a severely restricted lung function at diagnosis (average FVC 38% of predicted), a reduced diffusion capacity for CO and a marked desaturation on exercise, however were normocapnic (Additional file 1: Table S4).

### Radiology

**Figure 1:**
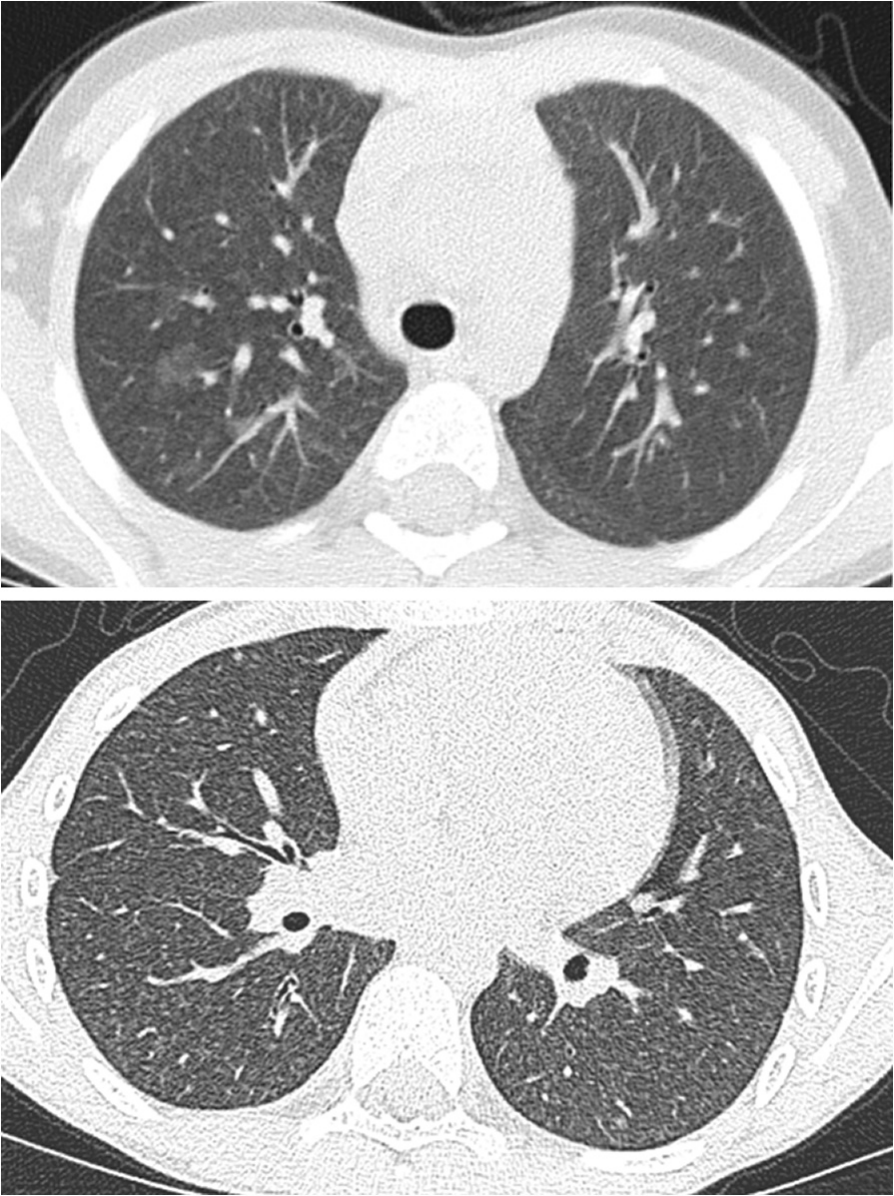
**HRCT findings of HP.** Upper panel shows acute HP with ground-glass opacification more prominent on the right side, the lower panel shows diffuse micronodules and ground-glass attenuation in subacute HP.

96% of the CT scans showed characteristic nodular opacities, 75% linear opacities, and 73% a ground glass pattern with increased attenuation (Figure 1). Except for reticular opacities which were present in 63% of all cases, other abnormalities were much less frequent (Table 2). The chest x-rays were scored first and in a blinded manner. Unexpected the frequency of the abnormalities were comparable to those scored on the CT scans.

**Table 2:** Results of blinded, independent scoring by two specialized radiologists of 30 chest x rays films taken in 16 children and 13 CT scans taken in 13 different children

	**Chest x-ray (p.a.)**		**Chest CT**	
**Abnormality present**	**Yes/total scored**	**% positive (mean (range))**	**Yes/total scored**	**% positive (mean (range))**
Hilar lymph nodes	6/30	20 (0–23)	2/13	12 (8–15)
Linear opacities	29/30	75 (53–97)	11/13	75 (65–85)
Reticular opacities	29/30	91 (85–97)	10/13	63 (50–77)
Nodular opacities	28/30	83 (73–93)	13/13	96 (92–100)
Cystic opacities	1/30	3 (2–3)	0/13	0 (0)
Bronchiectasis	5/30	10 (3–17)	1/13	8 (8–15)
Ground glass pattern, increased attenuation	24/30	72 (63–80)	10/13	73 (69–77)
Emphysema, reduced attenuation	0/30	0 (0)	0/13	0 (0)
Consolidation	4/30	12 (10–13)	0/13	10 (0–19)

The quality of the CT scans was judged as good in 9 cases, moderate in 3 and poor in 1. All were inspiratory scans, in four contrast medium was used, in six high resolution scans were available.

### Histology

**Figure 2:**
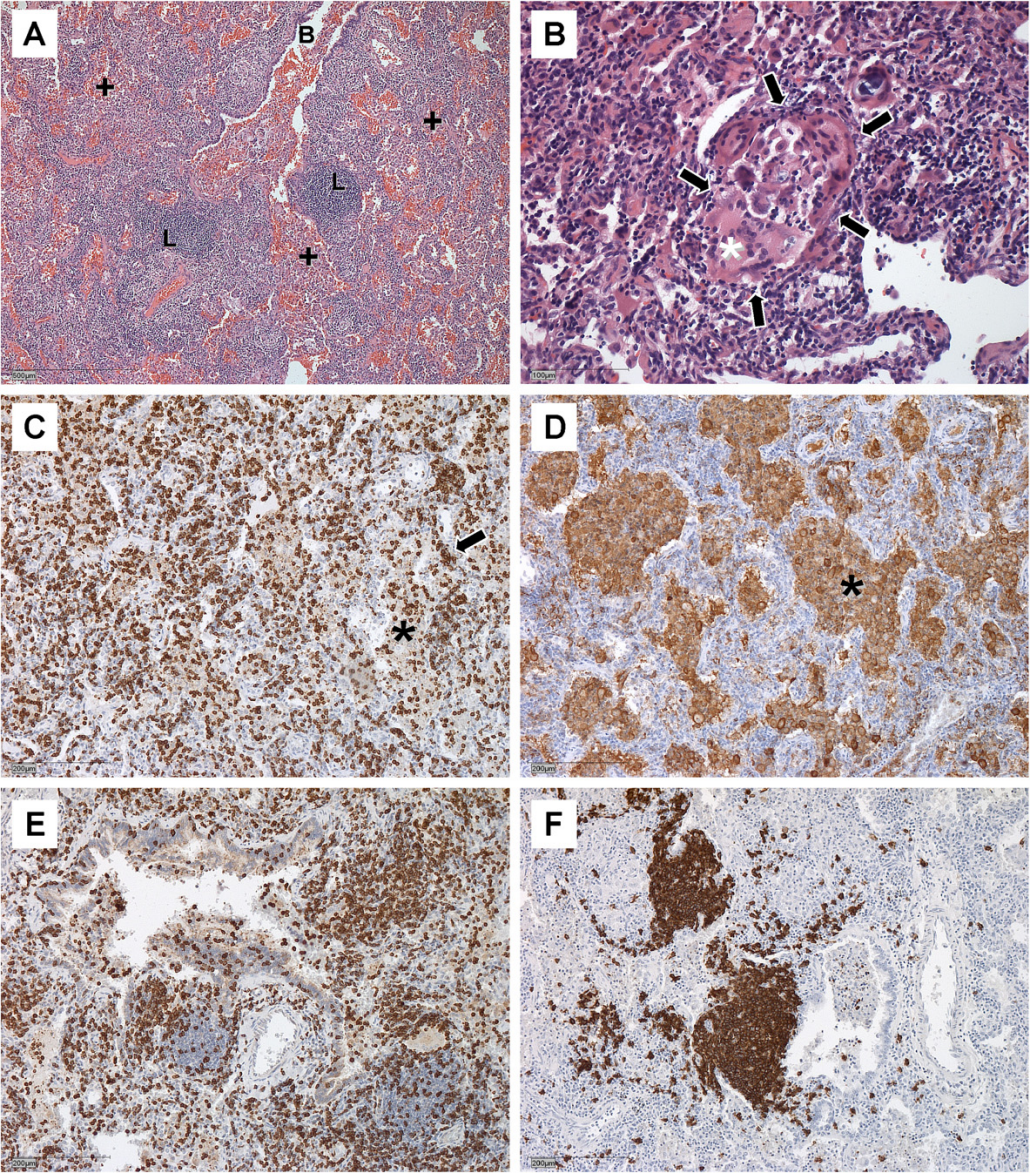
**The 3 typical findings of HP are 1. bronchiolitis, 2. alveolitis and 3. loosely formed non-caseating histiocytic granulomas. (A)** shows bronchiolitis (B) with lymph follicles (L) and increased amounts of lymphocytes in the walls of the bronchiole and alveolitis (+). Figure 2**(B)** depicts a typical granuloma (arrows) with multinucleated histiocytic giant cells (*). Immune histochemical stains for CD 3 confirm the presence of increased amounts of T cells in the alveoli (asterix) and septi (arrow) **(C)**, and stains CD68 the presence of macrophages in the alveoli (asterisk) **(D)**. **(E)** demonstrates intraepithelial T lymphocytes in the bronchial epithelium and **(F)** aggregates of B lymphocytes (lymph follicles). Single scattered B lymphocytes were also found in alveolar septa and alveoli. Magnification is indicated by bar in each figure.

In 6 of 23 children an open lung biopsy was done, as the diagnosis was not made on clinical and radiological grounds. Average age of diagnosis was not significantly lower than in the other children (Additional file 1: Table S3). In all patients, a mild to strong inflammation was found. Alveolar septal spaces were thickened by increased amounts of lymphocytes (Figure 2A) mainly T-lymphocytes (Figure 2C). Alveoli were variably filled with lymphocytes (Figure 2A), which were mainly T-lymphocytes (Figure 2E) and macrophages (Figure 2D). In three out of six cases loosely formed non-caseating histiocytic granulomas with multinucleated histiocytic giant cells (Figure 2B) were found. Furthermore a mild to strong bronchiolitis (Figure 2A) with many lymphocytes (mainly T-lymphocytes (Figure 2E)) and lymph follicles (mainly B-lymphocytes (Figure 2F)) in the bronchiolar wall as well as and many intraepithelial lymphocytes (mainly T-lymphocytes (Figure 2E)) was found.

### Treatment and outcome

All children but one were hospitalized for prolonged times, the average stay being 16 days. During this time all except 3 of the 23 children, were treated with systemic steroids. Only two centers did not use steroids (Table 3 and Additional file 1: Table S5). The systemic steroids were tapered over prolonged periods, on average given for almost 4 months. About 50% of the children in addition received inhaled corticosteroids. Of interest, even initial allergen avoidance was not strict in all children. In these, as well as in those children with allergen re-exposure, a prolonged course was observed and in one child the course worsened during the follow up of about 1 year after the initial diagnosis.

**Table 3:** Treatment and follow up of the 23 children with hypersensitivity pneumonitis included into the study

	
Empiric antibiotic therapy for suspected atypical pneumonia, response to this therapy	16 of 23 and 3 of 16
Allergen avoidance recommended and definitely eliminated	18 of 23
Initial stay in hospital (days)	16 ± 12
Systemic steroids	20 of 23
Dose of Prednisolone (mg/kg KG)	2 ± 0.5
Duration of taper (d)	119 ± 85
Inhaled steroids	11 of 23
Dose of budesonide equivalents (μg/d)	427 ± 271
Duration (weeks)	34 ± 22
Outcome	17 healthy
	5 improved
	1 worse
Follow up time (years)	1.1 ± 1.0

Data are given as absolute numbers or mean ± SD.

## Discussion

From this work 3 main conclusions can be derived; (1) diagnosis of pediatric HP is frequently late, despite characteristic symptoms at presentation, (2) allergen avoidance as the principal treatment is not always followed very strictly, and (3) prolonged and high dose steroid treatment is often used.

Although HP is the most frequent chronic interstitial lung disease in children [13], a quarter of the children with HP presented here and incident during a 3 y period in Germany, were diagnosed in chronic disease state. This together with the fact that lung biopsies were done at a relatively high frequency, suggests that some difficulties have to be overcome to diagnose this condition in childhood. All children, except one who was not measured, but was hypoxemic at rest (O2-saturation 80%), presented with a severely (≤65% pred.) restricted lung function and almost all had reduced oxygen saturation at rest or with exercise (Additional file 1: Table S3). These findings are very characteristic for presenting HP [17], nevertheless in 70% of the children an atypical pneumonia was diagnosed and empiric antibiotic treatment was started. Lung biopsy helped in all 6 instances when performed to substantiate the diagnosis and showed in all cases variable mural and luminal lymphocytic alveolitis as well as bronchiolitis, whereas loosely formed non-caseating histiocytic granulomata with multinucleated giant cells where found only in three cases; although in retrospect, diagnosis might have been possible from the combination of characteristic clinical features including the presence of serum precipitins, BAL lymphocytosis (except for 1 case), and FVC below 50% pred.

CT scans have recently been demonstrated to significantly contribute to the initial diagnosis of HP in adults, with specificity of 81% [5]. Their value in children has not yet been demonstrated. In this series in only 13 of 23 children CT’s were done, i.e. a diagnosis was reached in more than 40% without a CT scan. In addition, a review of the CT scans obtained demonstrated studies of variable quality. Only 9 of 13 cases with CT had a quality judged as good, and the quality was moderate in 3 and poor in 1. Major reasons for the failure were artifacts from respiratory motion. As already proposed for adults, these results highlight the need for and compliance with standardized protocols [18]. The characteristic radiologic features known from adult cases of HP were seen in these pediatric patients to similar extents [19]. Of interest was the high degree of concordance in the frequency of abnormal chest radiograph and CT findings. However it must be cautioned as only the chest CT findings “are characteristic” of HP.

The overwhelming majority of HP in the pediatric population is due to bird and fungus allergens; one of the challenges may be to identify alternative sources of these allergens, like fungus contaminated indoor hydroponics [3], misting fountains [20], basement showers [4] or possibly even wild city pigeons [21]. Up to 25% of the cases reported previously in the pediatric literature [5] and few additional case reports [2,3,22] were “familial” cases. Reasons may include (1) publication bias from preferring familial cases; (2) insensitivity of history to detect alone standing early disease, or (3) a higher likelihood for serologic testing in families with affected other members. Although common genetic predisposing factors may also be involved, they have not been demonstrated by now. Familiarity in the context of HP is most likely due to a common exposition to the antigens.

Identification of the responsible allergen is critical for avoidance and the causal and principal treatment [12]. Of interest, almost all children identified here were treated with systemic steroids. The advantage of such an approach is a more rapid therapeutic response with steroid treatment; however this introduces the impossibility to monitor the completeness of allergen avoidance measures. Lack of antigen avoidance and non-effectiveness of corticosteroid therapy results in pulmonary fibrosis and end-stage lung disease with death [23] or the need of lung transplant at young age [24]. Thus for treatment primarily careful allergen avoidance with associated clinical improvement is warranted. This proposition needs to be tested in a randomized controlled trial using steroids or placebo.

A weakness of the study may be that we recovered most, but not all incident cases, as one, published as a single case, and came to our attention during manuscript preparation [3]. Secondly, it is possible that some chronic forms might have been misdiagnosed as severe, steroid-resistant asthma [21]. Although there was a very high preponderance of male children (95%) in the pediatric cases reported until 2005 [5], evened out sex ratios were demonstrated recently in a population based study [25]. Therefore the male to female ratio of 9/14 found in the present study, suggests an almost homogenous sampling. Lastly, long term follow up was not possible with the design of the study, based on the German Surveillance Unit which is not prepared for longitudinal follow up. This can be achieved with register studies.

Among the strengths of the study is the relatively large cohort of contemporary and newly detected pediatric HP cases collected over a brief period of time. The observed current approach to diagnosis and therapy clearly demonstrates that the index of suspicion for HP needs to be increased substantially and that an early diagnosis must be established by much more quality controlled assessment of the clinical, radiological and laboratory findings. In particular, allergen avoidance is key for management. The role of corticoids which are generally used needs to be defined in prospective clinical trials.

These issues are surpassingly suited to be assessed in an international study of this rare entity. Capture and long term follow up of the widely scattered cases of HP in children occurring in remote places can be easily overcome by web-based studies within rare disease registries (http://www.kids-lung-register.eu). As such, the recently funded proposal “Orphans Unite: chILD better together – European Management Platform for Childhood Interstitial Lung Diseases” (http://www.childeu.net) under the FP7 program will provide an excellent base for this important task.

## Competing interests

The authors declare that they have no competing interests.

## Authors’ contributions

MG designed the study protocol, analyzed the data, and wrote the draft of the manuscript, and is the study guarantor, with full responsibility for the finished article, access to any data, and control of the decision to publish. MH and DH collected and analyzed the data, VT and JGP performed analysis of the imaging data, FB did the pathological analysis. MH, DH, VT, JGP and FB helped to write the manuscript. All the members of the National EAA study group contributed cases and supplied clinical data. All authors read and approved the final manuscript.

## Supplementary Material

Additional file 1: Table S1 Clinical presentation of the children. **Table S2.** Serum laboratory results. **Table S3.** Bronchoalveolar lavage and lung biopsy results. **Table S4.** Lung function. **Table S5.** Treatment and outcome. Click here for file

## National EAA Study Group

Tobias Ankermann, Children’s Hospital, University of Kiel, Germany; ankermann@pediatrics.uni-kiel.de

Katja Breuel, Children’s Hospital, University of Rostock, Germany; katja.breuel@med.uni-rostock.de

Achim Freihorst, Children’s Hospital, Aalen, Germany; Achim.Freihorst@ostalb-klinikum.de

Joern-Lorenz Gröbel, Klinikum Detmold, Germany; joern-lorenz.groebel@klinikum-lippe.de

Christoph Härtel, Children’s Hospital, University of Lübeck, Germany; haertel@paedia.ukl.mu-luebeck.de

Heinz Huprich, Children’s Hospital Datteln, Germany; H.Huprich@kinderklinik-datteln.de

Wolfgang Kamin, Children’s Hospital Hamm, Germany; sk-paediatrie@evkhamm.de

Joachim Lemke, Wilhelmstift Hamburg, Germany; j.Lemke@kkh-wilhelmstift.de

Frank Mandelkow, Kreiskrankenhaus Hagenow, Germany; f.mandelkow@krankenhaus-hagenow.de

Walther Mihatsch, Children’s Hospital, Schwäbisch Hall, Germany; walter.mihatsch@diaksha.de

Claus Pfannenstiel, Kinderarztpraxis Aachen, Germany; pfannenstiel@kinderarztpraxis-laurensberg.de

Michael Rau, Oberschwabenklinik, Ravensburg, Germany; michael.rau@Oberschwabenklinik.de

Johannes Schulze, Children’s Hospital, University of Frankfurt, Germany; Johannes.Schulze@kgu.de

Nicolaus Schwerk, Hannover Medical School, Germany; schwerk.nicolaus@mh-hannover.de

Jens-Oliver Steiß, Children’s Hospital, University of Gießen, Germany; Jens-Oliver.Steiss@paediat.med.uni-giessen.de

Heino Skopnik, Children’s Hospital Worms, Germany; paediatrie@klinikum-worms.de

Christian Teusch, München, Germany; c.teusch@me.com
