# Supplementary material for: Hypersensitivity pneumonitis: lessons for diagnosis and treatment of a rare entity in children
Source: Orphanet J Rare Dis. 2013 Aug 8;8:121. doi: 10.1186/1750-1172-8-121 (PMC3751081; doi:10.1186/1750-1172-8-121)
Supplement: Additional file 1: Table S1 — Clinical presentation of the children. Table S2. Serum laboratory results. Table S3. Bronchoalveolar lavage and lung biopsy results. Table S4. Lung function. Table S5. Treatment and outcome. [file 1750-1172-8-121-S1.doc]

Suppl-Table 1 Clinical presentation of the children

| Patient | Sex | Age at 1st visit (y) | Time to diagnosis (mon) | Presen-tation | Chronic cough | Dyspnoe at rest | Cyanosis  /Clubbing | Loss of weight per week until diagnosis | Other non-pulmonary diagnoses |
| --- | --- | --- | --- | --- | --- | --- | --- | --- | --- |
| 1 | f | 5.7 | 0.0 | a | - | yes | -/- | none | Atopic eczema (Dx at 5,58y) |
| 2 | m | 10.6 | 0.0 | a | - | yes | -/- | none | none |
| 3 | f | 14.3 | 3.0 | s | + | yes | -/- | 0.8 | Diabetes mellitus Typ I (Dx at 4 y), hyperthyreosis, adipositas, house dust mite allergy |
| 4 | f | 5.9 | 1.0 | c | + | no | +/+ | 0.1 | none |
| 5 | f | 11.6 | 0.0 | c | + | no | +/- | 1.0 | Atopic dermatitis (for years), adipositas, bronchial asthma (for years) |
| 6 | f | 8.0 | 1.0 | c | + | no | +/- | 1.0 | none |
| 7 | f | 13.4 | 1.0 | s | + | yes | +/- | 0.4 | Celic disease (Dx at 13 y), Small stature (Dx at 5,5 y) |
| 8 | f | 10.3 | 3.0 | s | + | no | -/- | None | none |
| 9 | m | 6.0 | 2.0 | s | + | yes | +/- | 0.1 | none |
| 10 | f | 7.8 | 2.0 | c | + | no | -/+ | 1.1 | none |
| 11 | f | 8.8 | 0.0 | a | nk | no | -/- | Nk | none |
| 12 | m | 10.6 | 2.0 | c | + | no | +/- | 0.2 | none |
| 13 | f | 11.4 | 0.0 | s | - | yes | +/- | None | Adipositas (Dx at 16 y) |
| 14 | m | 13.5 | 2 | c | + | yes | -/- | None | adipositas, hypothyroidism, allergy |
| 15 | m | 5.2 | 1.0 | s | + | yes | +/- | None | Alopecia areata, enuresis nocturna (Dx at 5y) |
| 16 | f | 9.9 | 1.9 | s | + | no | -/- | 0.6 | Vitiligo (Dx at 5y), celic disease suspected |
| 17 | m | 9.3 | 1.1 | s | - | no | -/- | 0.7 | None |
| 18 | m | 9.3 | 2.0 | a | - | yes | -/- | none | GERD and fundoplicatio at 3 y |
| 19 | m | 12.5 | 1.0 | c | + | yes | -/- | 1.2 | none |
| 20 | f | 15.1 | 1.1 | a | + | yes | -/+ | none | none |
| 21 | m | 11.3 | 0.0 | a | - | yes | +/- | 1.0 | none |
| 22 | f | 4.4 | 3.0 | c | + | no | +/- | none | none |
| 23 | f | 10.3 | 2.0 | c | - | yes | +/- | 1.2 | none |
| mean±SD | 9 m | 9.8±3 | 1.3±1 | a: 6  s: 8  c: 9 | 15 of 22 | 13 of 23 | 11 of 23  / 3 of 23 | 0.73 ± 0.49 |  |

Presentation: a: acute (within days to a week symptoms), s: subacute (symptoms for less than 4 weeks), c: chronic (symptoms for longer than 1 month).

Suppl-Table 2 Serum laboratory results

* removal of birds only from indoor; parents had commercial interest in birds

| Patient | Antigen clinically responsible for disease | Elevated fungus IgG | Elevated bird IgG | Allergen eliminated | Total IgG (fold upper limit) | LDH i.S. (U/l) | ACE i.S.(U/l) | Positive serology for mycoplasma | Antibiotic therapy for atypical pneumonia | Response to therapy |
| --- | --- | --- | --- | --- | --- | --- | --- | --- | --- | --- |
| 1 | Fungus, Bird | yes | yes | yes | 1.13 | 334 | 46 | no | Macrolide | nk |
| 2 | Bird | no | yes | yes | nk | nk | nk | nk | no | - |
| 3 | Bird | no | yes | yes | 1.11 | nk | 51 | no | no | - |
| 4 | Bird* | no | yes | no | nk | normal | 82.1 | yes | Macrolide | no |
| 5 | Down | no | yes | yes | nk | increased | nk | nk | no | - |
| 6 | Down | yes | yes | yes | nk | 420 | normal | yes | Macrolide | no |
| 7 | Fungus | yes | no | no | 1.15 | 399 | nk | no | Macrolide | yes |
| 8 | Bird, Down | yes | yes | yes | 0.91 | 338 | normal | yes | Macrolide | no |
| 9 | Bird, down | yes | yes | yes | 1.35 | 366 | 43 | no | no | - |
| 10 | Fungus | yes | yes | no | nk | nk | nk | no | Other antibiotics | no |
| 11 | Down | yes | yes | yes | nk | nk | 82 | nk | no | - |
| 12 | Down, bird | yes | yes | yes | 1.04 | nk | nk | yes | Macrolide | yes |
| 13 | Bird | yes | yes | yes | nk | normal | 35 | no | no | - |
| 14 | Down, Mold fungus | yes | yes | yes | 1.03 | 352 | 57 | yes | Doxycyclin | no |
| 15 | Bird | yes | yes | yes | nk | nk | nk | nk | Macrolide | no |
| 16 | Fungus, bird | yes | yes | yes | nk | 234 | 74 | yes | Macrolide | nk |
| 17 | Down | yes | yes | yes | nk | 479 | nk | yes | Macrolide | - |
| 18 | Bird | yes | yes | yes | 1.93 | nk | nk | no | Macrolide | yes |
| 19 | Bird | no | yes | yes | nk | nk | 20 | yes | Macrolide | no |
| 20 | Fungus | yes | yes | no | 1.10 | 204 | 32 | nk | no | - |
| 21 | not known | yes | no | yes | nk | 287 | 65 | nk | Macrolide | no |
| 22 | Down | yes | yes | no | 1.04 | 463 | normal | no | Macrolide | no |
| 23 | Bird | no | yes | yes | nk | nk | nk | yes | Doxycyclin | no |
| mean±SD |  | 17 of 23 | 21 of 23 | 18 of 23 | 1.2 ± 0.6 | 352 ± 189 | 53 ± 31 | 9 of 17 | 16 of 23 | 3 of 23 |

Suppl-Table 3 Bronchoalveolar lavage and lung biopsy results

| Patient | Total cell count (/μl)* | Macrophages (%) | Lymphocytes (%) | Neutrophils (%) | Eosinophils (%) | CD4+/CD8+ (% Lymph) | CD4/CD8 Ratio** | Cultered bacteria | Mykoplasma/Chlamydia PCR in BAL | Lung  biopsy |
| --- | --- | --- | --- | --- | --- | --- | --- | --- | --- | --- |
| 1 | Not done |  |  |  |  |  |  |  |  | no |
| 2 | 53000 | 48 | 47 | 1 | 1.3 | 58/5 | 12 | SA | - | no |
| 3 | Low | 95.3 | 4.7 | - | - | - | - | no | - | no |
| 4 | Nk | 53 | 23 | 32 | 2 | 55/44 | 1.25 | no | - | no |
| 5 | Nk | - | - | - | - | - | - | no | - | no |
| 6 | High | 42 | 55 | 2 | 1 | - | - | IF A+B | - | no |
| 7 | 360 | 12 | 36 | 36 | - | 22/70 | 0.31 | no | Neg/neg | yes |
| 8 | Nk | 48 | 30 | 17 | 5 | - | 0.8 | no | neg/neg | yes |
| 9 | Nk | 75.6 | 2.9 | 20.2 | 1.2 | - | - | SA. SP. HI | - | yes |
| 10 | Nk | 28 | 54 | 14 | 4 | - | 1.1 | no | -/neg | no |
| 11 | Nk | 17 | 80 | 2 | - | 35/58 | 0.6 | - | - | no |
| 12 | High | 60 | 10 | 30 | - | - | - | SA | Neg/- | no |
| 13 | 120 | 20 | 67 | 5 | 8 | 36/46 | 0.8 | SA | neg/neg | no |
| 14 | 1040 | 8 | 83.4 | 3.6 | 3.2 |  |  | no | neg/neg | no |
| 15 | Nd |  |  |  |  |  |  |  |  | yes |
| 16 | Nk | 26 | 65 | 6.7 | 0.4 | - | - | no | -/neg | yes |
| 17 | Nk | 31.2 | 31.4 | 32.6 | 0.8 | - | - | - | - | no |
| 18 | 4100 | 44 | 53 | 2 | 1 | 28/31 | 0.87 | no | neg/neg | yes |
| 19 | Not done |  |  |  |  |  |  |  |  | no |
| 20 | Nk | 36 | 57 | 3 | 4 | - | - | - | - | no |
| 21 | Nd |  |  |  |  |  |  |  |  | no |
| 22 | 82 | 68 | 47 | 13 | 2 | 17/79 | 0.21 | no | neg/neg | no |
| 23 | 2900 | 20 | 74.4 | 3.2 | 1.8 | - | - | SA | - | no |
| mean±SD | 8800±11017 | 41±25 | 46±26 | 13±13 | 2.6±2.1 | 36±20 | 2.0±2.8 |  | 7 neg of 7 | 6 of 23 |

SA: Staph aureus, IF: influenza, HI: Hämophilus influenzae

* normal < 13000/µl ; ** normal 0.7 ± 0.4 (range 0.1 to 1.9).

Suppl-Table 4 Lung function

| Patient | FEV1 (% pred) | FVC (% pred) | MEF25 (% pred) | DLCOcHb (% pred) | SaO2 (%)  Rest/excercise | pO2 (mmHg)  Rest/excercise | pCO2 (mmHg)  Rest/excercise |
| --- | --- | --- | --- | --- | --- | --- | --- |
| 1 | 30 | 31 | 66 | n.k. | 94/78 | n.k. /n.k. | n.k. /n.k |
| 2 | 68 | 63 | 46 | 63 | 96/94 | 79/67 | 34/31 |
| 3 | n.k. | 48 | n.k. | n.k. | 93/81 | 57/n.k. | 30.7/n.k |
| 4 | 40.1 | 35.2 | 140.4 | n.k. | 98/82 | 75/n.k. | 45/n.k |
| 5 | 40 | 34 | 63 | 35 | 95/92 | 65/n.k. | 42/n.k |
| 6 | n.k. | n.k. | n.k. | n.k. | 80/n.k. | n.k. /n.k. | n.k. /n.k |
| 7 | 33.9 | 21.4 | 30.3 | n.k. | 83/n.k. | 68.1/n.k. | 39.6/n.k |
| 8 | 50 | 48 | 42 | 52 | 95/92 | n.k. /n.k. | n.k. /n.k |
| 9 | 36 | 31 | 70 | n.k. | 98/79 | 68/n.k. | 37/n.k |
| 10 | 85 | 38 | n.k. | 86 | 98/87 | 61/n.k. | 36/n.k |
| 11 | n.k. | 33 | n.k. | n.k. | n.k./85 | n.k. /n.k. | n.k. /n.k |
| 12 | 58 | 65 | 58 | 31.9 | 98/88 | 44/n.k. | 48/n.k |
| 13 | 31 | 34 | 14 | 53 | 88/81 | 35/n.k. | 38.5/n.k |
| 14 | 35 | 30 | 31 | n.k | 93/70 | 75/68 | 34.7/38.1 |
| 15 | 25.9 | 22.04 | n.k. | n.k. | 90/n.k. | n.k. /n.k. | n.k. /n.k |
| 16 | 27.1 | 24.1 | 43 | n.k. | 96/80 | 76/n.k. | 36/n.k |
| 17 | 43 | 26 | 135.9 | n.k. | 97/n.k. | n.k. /n.k. | n.k. /n.k |
| 18 | 40.8 | 40.3 | 25.9 | 60 | 99/88 | n.k. /n.k. | n.k. /n.k |
| 19 | 41.7 | 34.7 | 155.6 | 39.2 | n.k. /n.k. | n.k. /n.k. | n.k. /n.k |
| 20 | 58.2 | 58.2 | 36.3 | 51.1 | 98/91 | 70/n.k. | 38/n.k |
| 21 | 58 | 53 | 60 | 45.1 | 93/n.k. | n.k. /n.k. | n.k. /n.k |
| 22 | 34 | 30 | n.k. | n.k. | 97/68 | n.k. /n.k. | n.k. /n.k |
| 23 | 50.8 | 45 | 35.1 | n.k. | 75/n.k. | 63/36 | 34.6/34.4 |
| mean±SD | 44±21 | 38±15 | 62±45 | 52±28 | 93±27/83±40 | 64±34/57±20 | 38±20/34±12 |

Suppl-Table 5 Treatment and outcome

| Patient | Allergen avoidance | Initial stay in hospital (days) | Syste-mic steroids | Dose of Prednisolone (mg/kg KG) | Dura-tion of taper (d) | Inhaled steroids | Dose of budenoside equivalents (μg/kg KG) | Duration (weeks) | Outcome | Remarks | Follow up time (years) |
| --- | --- | --- | --- | --- | --- | --- | --- | --- | --- | --- | --- |
| 1 | yes | 10 | yes | 2 | 210 | yes | 100 | 72 | healthy |  | 1.50 |
| 2 | yes | 20 | yes | 2 | 90 | no |  |  | improved | Re-exposition | 0.25 |
| 3 | yes | 15 | yes | 2.4 | 69 | no |  |  | healthy | Drop of precipitins after a year | 1.33 |
| 4 | no | 24 | yes | 1 | 60 | yes | 500 | 28 | healthy | No symptoms. but not completely free of exposition as birds only moved to outdoors | 1.75 |
| 5 | yes | 5 | yes |  | weeks | yes |  |  | improved | Persistent asthma symptoms after removal of allergens | 0.92 |
| 6 | yes | 13 | no |  |  | no |  |  | improved | Free of symptoms, but FVC ~ 80% of predicted | 2.00 |
| 7 | no | 43 | yes | 2 | n.k. | no |  |  | improved | Not completely recovered at last follow up | 0.25 |
| 8 | yes | 9 | yes | 2 | 270 | no |  |  | healthy |  | 0.75 |
| 9 | yes | 3 | yes | 2 | 240 | no |  |  | healthy |  | 0.92 |
| 10 | no | 26 | yes | 2 | 120 | yes | 800 | 24 | healthy |  | 0.83 |
| 11 | yes | 0 | yes | 2 | 90 | yes |  | 12 | healthy |  | 0.58 |
| 12 | yes | 10 | yes | 2 | 90 | yes |  | 28 | healthy |  | 0.83 |
| 13 | yes | 28 | yes | 2 | 235 | yes | 260 | 60 | healthy | Treatment induced Cushing-syndrome | 5.00 |
| 14 | yes | 21 | no |  |  | no |  |  | healthy |  | 0.75 |
| 15 | yes | 31 | yes | 1 | 42 | no |  |  | healthy |  | 1.67 |
| 16 | yes | 40 | yes |  | 90 | yes |  |  | healthy |  | 0.83 |
| 17 | yes | 7 | no |  |  | no |  |  | healthy |  | 1.08 |
| 18 | yes | 6 | yes | 3 | 60 | no |  |  | healthy |  | 0.33 |
| 19 | yes | 12 | yes | 3 | 60 | yes |  | 24 | healthy |  | 1.25 |
| 20 | no | 3 | yes | 2 | 200 | no |  |  | improved | Relapse with re-exposition during holiday | 0.67 |
| 21 | no | 6 | yes | 1.58 | 40 | no |  |  | healthy |  | 0.42 |
| 22 | no | 31 | yes | 2 | 180 | yes | 500 | 51 | healthy |  | 0.75 |
| 23 | yes | 7 | yes | 2 | 2 | yes | 400 | 6 | worse | Birds removed late after 2nd crisis with cyanosis, lost of follow-up | 0.17 |
| Mean ± SD | 17 of 23 | 16±12 | 20 of 23 | 2±0.5 | 119±85 | 11 of 23 | 427±271 | 34±22 | 17 healthy. 5 improved. 1 worse |  | 1.1±1.0 |
